# A Systematic Review for the Design of *In Vitro* Flow Studies of the Carotid Artery Bifurcation

**DOI:** 10.1007/s13239-019-00448-9

**Published:** 2019-12-10

**Authors:** A. M. Hoving, E. E. de Vries, J. Mikhal, G. J. de Borst, C. H. Slump

**Affiliations:** 1 University of Twente, 7500 AE Enschede, The Netherlands; 2 University Medical Center Utrecht, 3584 CX Utrecht, The Netherlands

**Keywords:** Model, Design, Imaging techniques, MRI, Optical PIV, Ultrasound

## Abstract

**Purpose:**

*In vitro* blood flow studies in carotid artery bifurcation models may contribute to understanding the influence of hemodynamics on carotid artery disease. However, the design of *in vitro* blood flow studies involves many steps and selection of imaging techniques, model materials, model design, and flow visualization parameters. Therefore, an overview of the possibilities and guidance for the design process is beneficial for researchers with less experience in flow studies.

**Methods:**

A systematic search to *in vitro* flow studies in carotid artery bifurcation models aiming at quantification and detailed flow visualization of blood flow dynamics results in inclusion of 42 articles.

**Results:**

Four categories of imaging techniques are distinguished: MRI, optical particle image velocimetry (PIV), ultrasound and miscellaneous techniques. Parameters for flow visualization are categorized into velocity, flow, shear-related, turbulent/disordered flow and other parameters. Model materials and design characteristics vary between study type.

**Conclusions:**

A simplified three-step design process is proposed for better fitting and adequate match with the pertinent research question at hand and as guidance for less experienced flow study researchers. The three consecutive selection steps are: flow parameters, image modality, and model materials and designs. Model materials depend on the chosen imaging technique, whereas choice of flow parameters is independent from imaging technique and is therefore only determined by the goal of the study.

## Introduction

Atherosclerotic plaque formation in the carotid artery bifurcation causes narrowing of the artery (stenosis) and the plaque may rupture, which can cause stroke or transient ischemic attack (TIA). Several parameters are known to influence risk of stroke in patients with significant carotid artery stenosis, e.g. plaque vulnerability, volume and stenosis degree.^20^,^26^,^52^ Also, there is an association between low and oscillating wall shear stresses (WSS) and formation and/or progression of atherosclerotic plaque.^19^,^41^,^57^,^76^ Surgical treatment is indicated in severe symptomatic carotid artery stenosis. An alternative approach is stenting of the lesion. However, this approach is not optimized yet, since it results in higher short-term stroke risk compared to surgery. There is a need for a better understanding of the factors that influence plaque characteristics and for analysis of flow changes caused by intervention, to eventually improve treatment and stroke prevention.

Blood flow studies are excellent approaches to enhance knowledge on the relationship between blood flow dynamics and plaque formation/progression and treatment outcome. General technological development leads to improvements in imaging and postprocessing techniques, which enables quantitative and detailed blood flow studies, such as image velocimetry. These techniques are superior to flow measurement techniques that only enable qualitative investigations, such as the way ultrasound Doppler is generally used in the clinic, namely only the measurement of flow in the center of the artery.

There are three methods to perform hemodynamic flow studies: *in vivo*, *in vitro* and *in silico*. The benefit of *in vitro* and *in silico* over *in vivo* is that certain parameters can be altered in a controlled environment. Compared to *in silico, in vitro* studies are sometimes preferred, due to the possibility to test and validate potential use of flow imaging techniques in patient studies. Furthermore, *in vitro* studies can be performed in situations where it is difficult to experiment on patients, for example in cases with radiation exposure or in case of rare diseases. Therefore, this review focusses on *in vitro* flow studies.

Starting *in vitro* flow studies brings along many steps and choices. For example, which imaging technique to choose from the wide range of (clinical) imaging modalities, to measure WSS or other flow parameters, and which phantom material to use. The choices for the flow setup also have to match the clinical research question. Review articles about *in vitro* flow study techniques often focus on one specific technique. Therefore, the aim of this review article is to give an overview of the possibilities of the various approaches for the design of *in vitro* flow studies. It will serve as guidance by best practice for researchers with less experience in flow studies to get familiar with the options and opportunities in flow study design.

## Materials and Methods

### Search Strategy

A systematic literature search was performed in January 2017 and repeated in June 2018. The key words are combinations of ‘carotid’, ‘flow’, ‘modeling’, ‘*in vitro*’, and synonyms of these terms, such as ‘set-up’/‘setup’, ‘blood flow velocity’, ‘rheology’, ‘wall shear stress’, ‘hemodynamics’, ‘simulation’, ‘phantom’. We have modified the search query to match each specific database (Scopus, Medline, Embase, and Cochrane).

### Study Selection

Two authors—A.H. and E.V.—independently screened the query results on the basis of titles and abstracts. Both authors independently checked full-text eligibility. All discrepancies regarding inclusion or exclusion were discussed until consensus was reached. The inclusion criteria were: (1) *in vitro* flow study; (2) carotid bifurcation models; and (3) quantitative flow imaging. The inclusion was limited to carotid bifurcation models, since the design of an *in vitro* flow study strongly depends on specific flow rates and vessel wall properties. Studies aiming at flow quantification in other arteries, for example abdominal aorta, might use other methods and characteristics, and are therefore not included. Thus, studies regarding intracranial carotid artery were excluded, as well as *in vivo* and animal studies. Also, studies using ultrasound Doppler measurement without further post processing and studies describing flow velocities only were excluded. Other exclusion criteria were: only *in silico*/computational fluid dynamics, full-text not in English, review articles and conference proceedings.

### Data Processing

The included full-text articles were organized into four categories of imaging techniques used to visualize flow: magnetic resonance imaging (MRI), laser particle image velocimetry (PIV), ultrasound, and miscellaneous techniques. Data extraction parameters for all imaging techniques are: resolution, study type (technique development/validation, flow exploration), working fluid, fluid scatters, flow type (steady, sinusoidal, physiologic), Reynolds number, viscosity, flow rate, velocity, model materials, model design: pathology (healthy, stenosed or aneurysmatic), geometry (average or patient-specific), wall (thin-walled or wall-less), origin (commercial or home-made); and flow visualization parameters. Technique-specific data extraction parameters were:

- for MRI: sequence;
- for optical PIV: light source;
- for ultrasound: protocol/postprocessing, system type (clinical or research);
- for miscellaneous techniques: methods.

## Results

**Figure 1:**
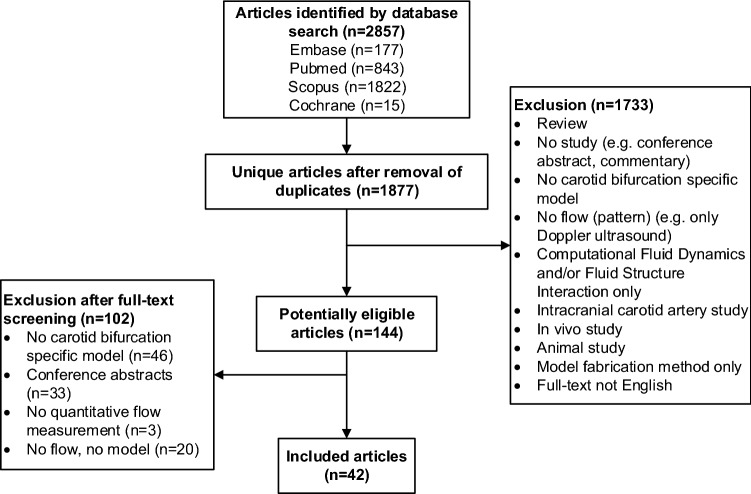
Schematic overview of systematic search.

The systematic search yields 1877 unique articles. Most articles are excluded on the basis of title and abstract screening. Full-text review includes 144 articles, of which 42 articles are selected for this review (Fig. 1).

**Figure 2:**
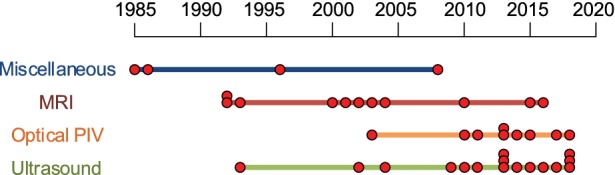
Timeline of all included articles. Each red dot shows one publication.

The oldest techniques to quantify blood flow patterns *in vitro* in carotid artery bifurcation that are included in this review, use laser doppler anemometry^34^ and digital subtraction angiography.^74^ The first articles that reported the use of MRI date from 1992. Studies using ultrasound or optical PIV techniques are mostly from 2008 and later. A timeline of included articles per imaging technique is shown in Fig. 2.

### Characteristics of Imaging Techniques and Methods

#### MRI

MRI is used in 14/42 articles (33%) to scan the *in vitro* carotid bifurcation model to visualize the flow (Table 1). One of these articles describes the use of both MRI and ultrasound.^18^ Among the several imaging sequences (MRI protocols) that are used, phase contrast sequences are applied in most articles.^4^,^11^,^18^,^33^,^38^,^39^,^43^,^46^,^54^,^56^,^78^ A distinction is made between two-dimensional and three-dimensional phase contrast sequences. Phase contrast MRI makes use of the phase shift of moving spinning protons. Velocity data can be computed by comparing the phase shifts between moving and stationary protons.

**Table 1:** Characteristics of MRI for *in vitro* flow studies.

Author	Sequence	Resolution	Study type	Working fluid	Flow type	Re number	Viscosity	Flow rate (mL/s)	Velocity (cm/s)
Napel 1992^43^	PC 3D	Th: 0.7	T	–	St	–	–	7.1	0–40*
Wolf 1992^66^	FT GRE 2D	Th: 5	T	Paramagnetically doped methylcellulose solution	Pu	–	0.006 Pa.s	10	−3 to 39
Frayne 1993^18^	PC 3D	S: 0.5 × 0.5 × 0.5	T–P	Machine tool cutting fluid, water	St	400	0.04 Pa.s	9	–
Vu 1993^65^	AFP	Th: 20	T	–	St	2219 (Ms)	10.07 × 10^−6^ m^2^/s	–	14.9
Botnar 2000^4^	PC	S: 0.5 × 0.5	T	Glycerin, water	Ph	488	37 × 10^−6^ m^2^/s	M: 9.4, P: 28	–
Kohler 2001^33^	PC 3D	–	T	–	St	–	0.003 Pa.s	10	–
Long 2002^38^	PC 3D	S: 0.63 × 0.63 × 0.8	T	BMF	St	330		10	–
Papathana-sopoulou 2003^46^	PC 3D	S: 0.51 × 0.51 × 1.4	F	–	Ph	–	0.0038 Pa.s	M: 7.2, P: 23.6	–
Zhao 2003^78^	PC 3D	S: 0.51 × 0.51 × 1.05	T	BMF-S	Ph	374	3.7 × 10^−6^ m^2^/s	M: 8.7, P: 19.7	–
Marshall 2004^39^	PC 3D	S: 0.5 × 0.5 × 1.4 Te: 50	T	BMF-S	Ph	–	0.0034 Pa.s	M: 7.2, 2-21*	–
Carvalho 2010^8^	CINE spiral FVE	Te: 23.2	T	–	–	–	–	–	–
Rispoli 2015^54^	PC 3D	S: 0.5 × 0.5 × 1.0 Te: 91.2	T	–	–	–	0.005 Pa.s	–	0-45*
Seong 2015^56^	PC	S: 0.247 × 0.247 × 1	F	Glycerin, water	Ph	666	3.6 × 10^−3^ Pa.s	M: 6, P: 12	–
Cibis 2016^11^	PC 2D	Several	T	–	Ph	–	0.001 Pa.s	0–9*	–

*2D* 2 dimensional, *3D* 3 dimensional, *BMF* blood-mimicking fluid, *BMF-S* blood-mimicking fluid (Shelley), *CINE spiral FVE* Cine(ma) spiral Fourier velocity encoding, *F* flow exploration, *FT GRE* Fourier transform Gradient Recalled Echo, *M* mean, *P* peak, *PC* phase contrast, *Ph* Physiologic, *Pu* pulsatile, *S* Spatial resolution in mm, *St* Steady/constant, *T* Technique development/validation, *T–P* Technique development/validation–Phantom, *Te* Temporal resolution in ms, *Th* (slice) thickness in mm, *extracted from figure, “−” NA

Three articles use other MRI-sequences to visualise flow. The first article proposes an extension of the single-bolus multi-zone adiabatic passage technique.^65^ This extended version uses flow velocity profiles from several directions other than the main flow direction. The second article describes the feasibility of spiral Fourier velocity encoded MRI for measuring carotid wall shear rate.^8^ Compared to standard Fourier velocity encoding, spiral Fourier velocity encoding is faster due to a higher temporal resolution, so wall shear rate can be measured not only *in vitro*, but also *in vivo*. The third article describes disordered flow, which can be visualised using temporal variations in magnetization by applying a two-dimensional Fourier Transform Gradient Echo sequence.^66^ The resulting images only show disordered flow.

#### Optical Particle Image Velocimetry

An alternative imaging technique to visualize flow is optical PIV. It is a technique that uses one or more lasers to illuminate contrast material flowing through a transparent phantom and captures the motion using a high-frame-rate digital camera. Each image frame is divided into small, so-called interrogation areas. Subsequently, each area is compared to the corresponding area of the following frame by applying correlation techniques. Finally, a velocity field is calculated, and localized velocity vectors can be visualized.

11/42 articles (26%) report on the use of optical PIV to study flow patterns in the carotid bifurcation in an *in vitro* model (Table 2). Two articles also use ultrasound.^59^,^77^ The flow setups are equipped with a continuous wave laser,^2^,^30^,^31^ a pulsed laser,^2^,^29^,^44^ or a LED light source.^40^ Where most of the studies uses one or two (stereo-PIV) cameras, one article describes the construction of a tomographic setup, using four digital cameras arranged at various angles.^7^

**Table 2:** Characteristics of optical PIV for *in vitro* flow studies.

Author	Laser type	Resolution	Study type	Working fluid	Fluid scatterers	Flow type	Re number	Viscosity	Flow rate (mL/s)	Velocity (cm/s)
Bale – Glickman 2003^2^	C&Pu	Te: 30	F	Isopropyl alcohol, glycerin	Silver coated hollow glass spheres	St	13, 185, 410	0.15 × 10^−6^ m^2^/s	–	P: 40& 49, D: 14&12
Cheung 2010^10^	–	S: 0.13 × 0.13	F	Glycerin, water	Rhodamine B fluorescent particles	St	485	6.2 × 10^−6^ m^2^/s	P: 12.17	–
Buchmann 2011^7^	–	S: 1.6 × 1.6 × 1.6	T	Glycerin, water	Hollow glass spheres	St	339	12.7 × 10^−3^ Pa.s	–	Max: 37
Zhang 2011^77^	Pu	S: 0.5 Te: 1428	T	Dionized water	Cornstarch	Ph	1700	1.0 × 10^−6^ m^2^/s	15.5	P: 70
Kabinejadian 2013^29^	Pu	–	F	Glycerin, water	Polyamid particles	Ph	–	0.0055 Pa.s	M: 47	–
Kefayati 2013^31^	C	Te: 1000	F	Water, glycerin, sodium iodide	Rhodamine B-encapsulated microspheres	Ph	289	4.31 × 10^−3^ Pa.s	M: 6.3 P: 27	–
Kefayati 2014^30^	C	S: 0.3 × 0.3 Te: 1000	F	BMF^75^	Polymer fluorescent microspheres	Ph	312, 473, 789	4.31 × 10^−3^ Pa.s	M: 6.29 P: 27.13	–
Nemati 2015^44^	Pu	S: 0.3 × 0.3	F	Glycerin, water	Hollow glass balls	Ph	512	–	40	–
Mokthar 2017^40^	LED	Te: 120	F	Glycerin, water	Polyamid particles	St	–	1.587 × 10^−6^ m^2^/s	–	6
Shimizu 2017^59^	–	–	T	Polyethylene glycol	Glass particles	St&Si		0.006 Pa.s	–	0–25*
Hewlin 2018^24^	C	–	T-P	Water	Spherical hollow glass particulates	Ph	–	6.986 × 10^−7^**	Max: 17.10^3	Max: 45

*BMF* blood-mimicking fluid, *C* continuous laser, *D* diastole, *F* flow exploration, *M* mean, *Ms* measured, *P* peak, *Pu* pulsed laser, *Ph* Physiologic, *S* spatial resolution in mm, *Si* sinusoidal, *St* Steady/constant, *T* Technique development/validation, *T–P* Technique development/validation–Phantom, *Te* Temporal resolution in Hz, *Th* (slice) thickness in mm, *Extracted from figure, **Kinematic, no units mentioned, “–” NA

#### Ultrasound

In 16/42 articles (38%), ultrasound is applied as imaging technique (Table 3). Three research groups contribute to this review by two or more included articles. The applied equipment is almost similar within these groups. Both clinical and research-based ultrasound systems are used.

**Table 3:** Characteristics of ultrasound for *in vitro* flow studies.

Author	Group	Protocol/postprocessing	System type	Resolution	Study type	Working fluid	Additional Fluid scatterers	Flow type	Re number	Viscosity	Flow rate (mL/s)	Velocity (cm/s)
Lai 2013^35^	HK	PD DCF	R	–	T-P	BMF-S	–	Ph	242	3.95 × 10^−6^ m^2^/s	M: 4.5 P: 14	–
Yiu 2013^73^	HK	CESI	R	Te: 2000, S: 0.15 × 0.15	T	BMF-S	–	Ph	80	3.95 × 10^−6^ m^2^/s	M: 1.5 P: 5	M: 5.3, P: 35.4
Yiu 2014^72^	HK	SPW	R	Te: 416	T	BMF-S	–	Ph	–	3.95 × 10^−6^ m^2^/s	P: 5	0-80*
Chee 2016^9^	HK	DCF PWI	C&R	–	T-P	BMF-S	–	Ph	–	–	M: 1.95 P: 6.5	–
Leow 2015^36^,^37^	LO	PW-PI IV	R	Te: 1000	T	Glycerin, water	MB^58^	Ph	–	–	4	–
Leow 2018^37^	LO	PI SPW IV	R	–	T	Glycerin, water	MB^58^	Ph	–	–	–	0–40*
Poepping 2002^48^	RRI	D	C	Te: 83	T	BMF-R	–	Ph	104	4.1 × 10^−3^ Pa.s	M: 9 P: 20	–
Poepping 2004^49^	RRI	D	C	–	T-P	BMF-R	–	St	–	–	5	–
Poepping 2010^50^	RRI	PD	C	Te: 43	F	BMF-R	–	Ph	–	–	M: 5.1 P: 20	–
Wong 2009^68^	UWO	D	C	S: 1	F	BMF-R	–	Ph	238	–	M: 6.00 P: 23.46	–
Wong 2013^67^	UWO	D	C	Te: 83	F	BMF-R	–	Ph	–	4.01 × 10^−6^ m^2^/s	M: 6.00 P: 23.46	− 50 to 100*
Frayne 1993^18^	–		C	S: 0.17 × 0.14 × 0.58	T-P	Machine tool cutting fluid, water	Cellulose particles	St	400	0.04 Pa.s	9	–
Zhang 2011^77^	–	E-PIV	R	Te: 1428, S: 0.5	T	Water	MB	Ph	1700	1.0 × 10^−6^ m^2^/s	15.5	P: 70
Shimizu 2017^59^	–	D	C	–	T	Polyethylene glycol	Glass particles	St&Si	–	0.006 Pa.s	–	0 to 25*
Jensen 2018^28^	–	IV	R	S: 1.1 × 1.1	T	BMF-S	–	Ph	–	4.1 × 10^−3^ Pa.s	P: 15	–
Niu 2018^45^	–	IV	R	Te: 125	F	–	MB	Ph	–	–	–	–

*BMF* blood-mimicking fluid, *BMF-S* blood-mimicking fluid (Shelley), *BMF-R* US blood-mimicking fluid: water, glycerol, dextran, surfactant, nylon particles,^51^*C* clinical system, *CESI* Color-encoded speckle imaging, *D* Doppler, *DCF* Doppler Color Flow, *E-PIV* echo particle image velocimetry, *F* Flow exploration, *HK* published by research group at University of Hong Kong, *HM* home-made, *IV* image velocimetry, *LO* published by research group at London Imperial College, *M* mean, *MB* microbubbles, *P* peak, *Ph* Physiologic, *PD* pulsed Doppler, *PI* pulse inversion, *PWI* plane wave imaging, *PW-PI* plane wave pulse inversion, *R* research system, *RRI* published by Robarts Research Institute, *S* Spatial resolution in mm, *Si* sinusoidal, *SPW* steered plane wave, *St* Steady/constant, *T* Technique development/validation, *T–P* Technique development/validation–Phantom, *Th* (slice) thickness in mm, *Te* Temporal resolution in Hz, *UWO* published by research group at The University of Western Ontario, *Extracted from figure, “−” NA

Seven out of 16 articles within the ultrasound category perform image velocimetry. Different acquisition protocols and post processing techniques are used: echo particle image velocimetry using contrast agent (echoPIV),^77^ high-frame-rate ultrasound imaging velocimetry using speckle patterns,^36^,^37^ Vector Projectile Imaging using multi-angle Doppler analysis,^72^ transverse oscillation and directional beamforming for vector velocity estimation,^28^ a biomechanical method that produces a map of displacement vectors,^45^ and vector flow mapping using color Doppler images from a clinical system.^59^

Doppler protocols are applied in seven articles. One article uses a Doppler protocol to measure volume flow.^18^ Some articles describe the use of a semiautomatic Doppler ultrasound acquisition system to obtain small sample volumes at desired spatial intervals to perform velocity measurements over time.^48^^–^50,67,68 In another article, pulsed Doppler and color flow imaging are applied on a research system to investigate velocities in home-made phantoms.^35^

A combination of clinical Doppler flow measurements and an advance plane wave protocol on a research-based system is also described.^9^ Another technique applied in Ref. 73 called color-encoded speckle imaging, uses high-frame-rate steered plane wave imaging on a system that includes a pre-beamformed RF data acquisition tool (a channel-domain imaging research platform).

#### Miscellaneous Techniques

4/42 articles (10%) report miscellaneous techniques for flow visualization (Table 4). One article describes the use of digital photographic imaging in combination with photochromic dye.^12^ A novel grid reconstruction technique has resulted in development of quantitative measurements. The use of Laser Doppler Anemometry (LDA) is described in two articles.^14^,^34^ This technique is based on the Doppler shift induced by scattering of a laser beam when it hits moving fluid. Flow rate is measured using Digital Subtraction Angiography in the fourth article.^74^ Time density curves are created and blood flow among the regions of interest is calculated using the obtained velocity and known radius of the vessel.

**Table 4:** Characteristics of miscellaneous techniques for *in vitro* flow studies.

Author	Method	Resolution	Study type	Test fluid	Fluid contrast	Flow type	Re number	Viscosity	Flow rate (mL/s)	Velocity (cm/s)
Ku 1985^34^	LDA	–	F	Glycerin, water	–	Ph	300	12 × 10^−6^ m^2^/s	M: 5 P: 13.3	–
Yoshida 1986^74^	DSA	Te: 30	T	Water	Contrast medium	St	–	–	10, 16, 20	–
Couch 1996^12^	Photo-chromic grid	–	T	Deoderized kerosene	Photo-chromic dye	St	1200	1.8 × 10^−6^ m^2^/s	–	−10 to 66 *
Ding 2008^14^	LDA	–	F	Water, glycerin, sodium thiocyanate	Black ink	Ph	300	2.875 × 10^−6^ m^2^/s	3-26*	–

*DSA* digital subtraction angiography, *LDA* laser doppler anemometry, *F* Flow exploration, *M* mean, *P* peak, *Ph* Physiologic, *St* Steady/constant, *T* Technique development/validation, *Te* Temporal resolution in Hz, *Extracted from figure, “−” NA

### Model Characteristics

A full overview of the characteristics of the bifurcation models is presented in Table 5. Most of the MRI articles make use of a commercial model, in contrast to the majority of the optical PIV and ultrasound articles, where home-made models are used. Casting is a technique that is frequently used to obtain certain geometries in home-made models. For example, rapid prototyping or 3D printing techniques are used to retrieve molds and one article even describes a fabrication method for separate plaque inclusion in the model.^49^ Between groups of imaging techniques and within the groups, different materials are used for the fabrication of the home-made models. Most optical PIV models are made of silicone. In the ultrasound articles, polyvinylalcohol (PVA) is a frequently used material, especially in the last decade.

**Table 5:** Model characteristics of all included papers.

Author	Fabrication material	Pathology	Geometry	Wall	Origin
MRI					
Napel 1992^43^	–	–	–	–	–
Wolf 1992^66^	–	H & S	–	–	–
Frayne 1993^18^	Polyester resin + TMM (agar-based)	H	AG	TW	HM
Vu 1993^65^	Glass	–	AG	–	HM
Botnar 2000^4^	Silicone	H	PS	WL	HM
Kohler 2001^33^	(1) Plexiglass (Perspex) (2) –	(1) – (2) H	–	–	(1) HM (2) CM
Long 2002^38^	–	–	–	–	CM
Papathanasopoulou 2003^46^	–	H	–	–	CM
Zhao 2003^78^	Acrylic	H	–	–	CM
Marshall 2004^39^	–	H & S	–	–	CM
Carvalho 2010^8^	–	–	–	–	CM
Rispoli 2015^54^	–	H	–	–	CM
Seong 2015^56^	Silicone	H	AG	–	HM
Cibis 2016^11^	–	H	PS	–	HM
Optical PIV					
Bale – Glickman 2003^2^	Silicone	S	PS	WL	HM
Cheung 2010^10^	Silicone (Sylgard 184)	S	PS	WL	HM
Buchmann 2011^7^	Silicone (Sylgard 184)	S	PS	WL	HM
Zhang 2011^77^	Silicone	H	PS	TW	HM
Kabinejadian 2013^29^	Silicone (PDMS)	H	PS	WL	HM
Kefayati 2013^31^	Silicone (Sylgard 184)	H & S	AG	WL	HM
Kefayati 2014^30^	Silicone (Sylgard 184^50^)	H & S	AG	WL	HM
Nemati 2015^44^	Silicone (PDMS)	S	PS	WL	HM
Mokthar 2017^40^	Perspex	A	AG	WL	HM
Shimizu 2017^59^	Permeable urethane	–	PS	TW	HM
Hewlin 2018^24^	Glass	–	PS	TW	HM
Ultrasound					
Frayne 1993^18^	Polyester resin + TMM (agar-based)	H	AG	TW	HM
Poepping 2002^48^	Agar	S	AG	WL	HM
Poepping 2004^49^	(1) Silicone (Sylgard 184) + TMM (Agar-based) (2) Agar	S	AG	(1) TW (2) WL	HM
Wong 2009^68^	PTFE (Teflon)^69^	S & U	AG	WL	HM
Poepping 2010^50^	Silicone (Sylgard 184) + TMM (Agar-based^49^)	S	AG	TW	HM
Zhang 2011^77^	Silicone	H	PS	TW	HM
Lai 2013^35^	Compliant photopolymer + TMM (Agar-based)	H & S & U	AG	TW	HM
Wong 2013^67^	PTFE (Teflon)^69^	S & U	AG	–	HM
Yiu 2013^73^	PVA	S	AG	WL	HM
Yiu 2014^72^	–	H & S	AG	–	–
Leow 2015^36^	Compliant photopolymer + TMM (Agar-based)^35^	–	AG	TW	HM
Chee 2016^9^	PVA + TMM (Agar-based)	H & S	AG	TW	HM
Shimizu 2017^59^	Permeable urethane	–	PS	TW	HM
Jensen 2018^28^	PVA	H & S	PS	WL	HM
Leow 2018^37^	PVA	H & S	–	WL	HM
Niu 2018^45^	(1) – (2) PVA	H	AG	TW	(1) CM (2) HM
Miscellaneous					
Ku 1985^34^ (LDA)	Glass and plexiglass	H	AG	–	HM
Yoshida 1986^74^ (DSA)	Vinyl	H	AG	TW	HM
Couch 1996^12^ (Photochromic grid)	Plexiglass	H	AG	WL	HM
Ding 2008^14^ (LDA)	Glass	H	AG	TW^15^	HM

*LDA* laser doppler anemometry, *DSA* digital subtraction angiography, *PDMS* polydimethylsiloxane, *PVA* polyvinyl alcohol, *PTFE* polytetrafluoroethylene, *H* healthy, *S* stenosed, *A* aneurysmatic, *U*  ulceration, *PS* patient-specific, *AG* average geometry, *TW* thin-walled, *WL* wall-less, *HM* home-made, *CM* commercial model, “−” NA

Both healthy and diseased carotid artery bifurcations are studied. One of the models with diseased geometry is an aneurysmatic bifurcation,^40^ all other diseased models are stenotic, sometimes with ulceration.^35^,^67^,^68^ The geometries are based on either patient-specific geometries or averaged bifurcation dimensions. The home-made models are either ‘thin-walled’, meaning that the model has a certain (thin) wall thickness, or ‘wall-less’, that means that the fluid space is frequently surrounded by a block of material, instead of a thin wall. Thin-walled models surrounded by a tissue mimicking material (TMM) are also reported.^9^,^18^,^35^,^36^,^49^,^50^ The optical PIV articles mainly use wall-less models. One article describes the fabrication of a model that was compatible with x-ray, ultrasound and MRI.^18^ The article illustrated a polyester resin thin-walled model with a layer of agar as tissue-mimicking-material to be compatible with the different imaging techniques.

### Working Fluid, Scatter Particles and Contrast Agents

Flow studies in the human body can be performed without addition of scatter particles or contrast materials, in which case blood functions as natural contrast agent. However, for some techniques it is necessary to add contrast agents to the blood circulation, for example in some ultrasound protocols to enhance the scattering properties of blood. In case of *in vitro* studies, scatter particles or contrast material is regularly used, since the natural contrast enhancing properties of blood are not present.

No contrast agents or scatter particles are used in the MRI studies. The working fluid in MRI varies from commercially available blood mimicking fluid to an aqueous mixture of machine tool cutting fluid (Table 1). Optical PIV requires the addition of scatter material. In ten of eleven studies synthetic particles are added to the working fluid (Table 2). One study uses cornstarch as reflective material.^77^ There are several working fluids reported in the optical PIV studies, however, most studies use a glycerol-water mixture. Four of sixteen ultrasound studies use microbubbles as contrast material, both home-made^36^,^37^,^45^ and commercial^77^ (Table 3). Most other ultrasound articles use a blood mimicking fluid containing nylon particles.^9^,^28^,^35^,^48^^–^50,67,68,72,73 The commercial available BMF from Shelley Medical Imaging (BMF-S in Table 3) is based on the recipe of^51^ (BMF-R in Table 3). A photochromic dye is used in one article in the ‘miscellaneous’ category^12^ (Table 4). Another ‘miscellaneous’ article uses a mixture of 76% Renografin digital subtraction angiography contrast medium, sodium and meglumine to visualize flow using x-ray techniques.^74^

### Flow Visualization Parameters

Flow characteristics can be presented and visualized using many quantitative parameters, each accentuating different aspects of the blood flow. Some quantities need to be measured to estimate or calculate other, more relevant parameters. This review only includes articles that quantified and visualized flow characteristics and patterns.

The flow visualization parameters (Table 6) are divided into five categories: velocity, flow, shear-related, turbulent/disordered flow and other parameters. Velocity and flow are commonly used parameters and mostly presented as magnitude values, vectors, streak-/streamlines, and profiles over time. All imaging techniques are able to measure these two parameters. Wall shear stress (WSS) is a commonly calculated parameter in quantitative flow studies. Arterial WSS is defined as: “the drag force acting on the endothelium as a result of blood flow”.^8^ WSS magnitude is calculated by multiplying wall shear rate (WSR) by fluid viscosity,^8^,^11^ as shown in Ref. 33 with the following equation:

$$WSS = - \eta \left| {\frac{dv}{dr}} \right|_{r = a} ,$$

where *η* is the fluid viscosity, *v* is the velocity, *r* is the radial co-ordinate, and *a* is the vessel radius. Oscillating wall shear stress (OSI) (Table 6) can be calculated as follows:$OSI(\vec{s}) = 0.5\left[ {1 - \frac{{\left| {\sum\nolimits_{0}^{T} {WSS(\vec{s},t)\Delta t} } \right|}}{{\sum\nolimits_{0}^{T} {\left| {WSS(\vec{s},t)} \right|\Delta t} }}} \right]$,where $\vec{s}$ is the position at the vessel wall, *t* is the timepoint, Δ*t* is time step, and T is the number of time steps within one cardiac cycle.^11^,^23^

**Table 6:** Flow visualization parameters.

Parameter	Description/application	MRI	Opt PIV	US	Miscellaneous
Velocity					
(mean/peak) velocity	Often expressed in cm/s and can indicate pathologies, for example velocity increases in narrowed vessels	4^4^,^8^,^54^,^65^	2^30^,^77^	11^9^,^18^,^28^,^35^,^48^,^50^,^67^,^68^,^72^,^73^,^77^	1^34^
Velocity vectors/velocity field	Indicate both magnitude and direction	3^39^,^54^,^56^	10^2^,^7^,^10^,^24^,^29^,^31^,^40^,^44^,^59^,^77^	4^37^,^59^,^72^,^77^	1^12^
Secondary/circumferential/in plane velocity	Component of velocity orthogonal to largest velocity vector. Compare measurement and CFD results; indicate complex flow; indicate small flow disturbances	4^4^,^38^,^56^,^78^	3^7^,^10^,^31^	–	1^34^
Stream/streak lines	Show pattern of velocity or flow in phantom either in 2D or 3D. Provide information about blood flow disturbances	1^43^	5^2^,^7^,^29^,^31^,^77^	2^73^,^77^	1^12^
Velocity profile	Velocity magnitude over one axis through the model. Show differences in measurement and expectation; show differences in flow pattern on several positions; calculate shear stresses	3^38^,^66^,^78^	5^2^,^7^,^10^,^59^,^77^	5^28^,^48^,^49^,^59^,^77^	1^14^
Velocity waveform	Development of blood flow velocity over time. Show differences in measurement and expectation; define measurement accuracy; compare healthy and diseased models	–	1^77^	5^36^,^37^,^48^,^50^,^77^	–
Velocity contour	Velocity values in one cross-section of the model. Compare between different measurements	1^78^	3^7^,^30^,^44^	–	–
Velocity gradient	Compare measurement and CFD results, especially at the walls	–	1^59^	1^59^	–
Flow					
Flow	Volume of fluid per unit time	2^11^	–	–	1^74^
Flow vectors/patterns	Indicate both magnitude and direction of flow. Show complex flow or recirculation zones at specific timepoint or over a cardiac cycle	–	–	1^36^	–
Flow waveform	Show flow value in time at a specific position in the phantom. Indicate variations over time and compare measurements with golden standard	4^11^,^39^,^46^,^56^	2^31^,^77^	1^77^	–
Shear parameters					
Wall shear rate (WSR)	Defined as flow velocity gradient near the vessel wall^37^; used to calculate WSS; presented as: magnitude overlay on image; graph (magnitude over time, magnitude per position on wall)	1^8^	1^77^	3^37^,^45^,^77^	–
WSS magnitude	Show distribution of WSS in cross-sections or in graphs to show changes over time. In Pa or N/m^2^.	3^11^,^39^,^46^	4^2^,^7^,^30^,^59^	1^59^	–
WSS vectors/fields	Indicate both magnitude and direction. Show distribution of WSS in specific part of phantom in both 2D and 3D	3^33^,^39^,^46^	–	–	1^14^
Oscillatory shear index	Show wall shear stress fluctuations over time	2^8^,^11^	–	–	–
Reynolds shear stress	When shear stress is based on the fluctuating part of the velocity field (derived by Reynolds decomposition). Indicates vortices, areas of turbulent flow	–	1^30^	–	–
Stress phase angle	Temporal phase angle between WSS and circumferential strain. Plays an important role in arterial disorders	–	–	1^45^	–
Turbulent/disordered flow					
Turbulence (intensity)	Shows flow fluctuations over time. Quantifies fluctuations in flow not related to physiologic pulsatile flow	–	2^a,^^30^,^31^	4^48^,^50^,^67^,^68^	–
Standard deviation in peak velocity	Quantifies fluctuations in peak flow that are not related to physiologic pulsatile flow	–	–	1^68^	–
Swirling strength	Quantifies the strength of swirling motions by the imaginary part of the complex eigenvalues of the gradient tensor	–	2^a,^^30^,^31^	–	–
Disturbed flow overlay	Shows a coloured overlay of disordered flow measured by variation in phase signal on MRI	1^66^	–	–	–
Vorticity (fields)	Quantifies degree of vortexes in space or over time	–	2^2^,^10^	1^28^	–
Spectral-broadening index/spectral width	Indicates regions of recirculation, small flow values and spread associated within the velocity spectrum	–	–	4^35^,^48^,^50^,^68^	–
Doppler spectrograms	Indicates different levels of flow disturbances	–	–	2^a^^9^,^35^	–
Others					
Pulsatility index	Quantifies pulsatility of blood by calculation of difference between systolic and diastolic velocity	1^56^	–	–	–
Kinetic energy (KE)	KE can be seen as dynamic pressure in fluid stream. Changes in KE seem to play a role in arterial remodelling	1^56^	–	–	–

^a^From same research group

Most of the parameters describing turbulent or disordered flow are only reported by one paper or one research group and are thus measured using one visualization technique. Pulsatility index (PI) can be calculated following: $PI = \frac{{V_{\text{max systole}} - V_{\text{max diastole}} }}{{V_{\text{ave during cardiac cycle}} }}$. The same article defines kinetic energy (KE) as: $KE = \frac{1}{2}mV^{2}$.^56^

## Discussion

This systematic review serves as starting point for designing *in vitro* carotid flow studies by presenting an overview of methods that have been applied in *in vitro* hemodynamic studies using imaging techniques to visualize and quantify flow and flow-related parameters. The review is limited to research using carotid artery bifurcation models. The next paragraph shortly summarizes the results, followed by a discussion on considerations for *in vitro* flow studies, to be concluded with a section of strengths and limitations of this review.

### Summary of Results

We distinguished four categories of imaging techniques used to visualize and quantify blood flow dynamics: MRI, optical PIV, ultrasound, and miscellaneous techniques. A trend towards the use of optical PIV and ultrasound is seen in the last decade (Fig. 2). Noticeable in model design is the use of commercial models in MRI, while the other studies mainly use home-made models. Furthermore, the choice of model material depends on the imaging technique (Table 5). Optical PIV and ultrasound require the use of scatter particles, whereas the use of scatter particles in MRI studies is limited. Visualization parameters are divided into four categories (Table 6). Velocity-based parameters are widely reported, flow- and shear-based parameters are frequently studied, and turbulent/disordered flow-based parameters are mostly reported for optical PIV and ultrasound studies.

### Considerations for *In Vitro* Flow Study Design

The starting point of flow studies in general strongly depends on the goal of the study. Since there is a large variety of goals and aims in flow studies, the authors of this review article are not in the position to select one favourite flow parameter, imaging technique, model material or design for future use. This section illustrates a simplified three-step design process, combining and discussing the information from the results section.

The first step in flow study design is selection of parameters for flow visualization. This is strongly related to the goal of the study. Clinical relevance can also be considered while selecting flow visualization parameters. However, it does not restrict the imaging modality choice. Velocity and flow-based parameters give a global impression on flow dynamics and are presented in many ways: over time, spatial, mean or peak, as contour, etc. (see Table 6). Shear-based parameters are frequently studied, however their clinical relevance has yet to be determined. It is generally accepted that regions of low WSS and oscillating WSS correlate to formation and growth of atherosclerotic plaque.^53^,^57^ However, a review on this topic shows that there are novel studies that find an inverse relationship.^47^ Moreover, there is no clear conclusion about the relation between low and high WSS in plaque vulnerability.^8^,^30^,^70^ Turbulent/disordered flow-based parameters are mostly reported by ultrasound and optical PIV studies. Some of these parameters clearly show and quantify regions of disordered flow, however some other quantitative parameters are hard to interpret.

The second step in flow study design is selection of an imaging technique. Generally, the choice of visualization parameters does not lead to restrictions in imaging techniques, thus the three main imaging techniques (MRI, optical PIV, ultrasound) are all available after flow parameter selection. The benefits and limitations shown in Table 7 are discussed next. It is necessary to realize that some imaging techniques require the use of (clinical) contrast agents or scatter particles when using it *in vitro*. In this respect, MRI is beneficial, since retrieving flow information is not dependent on scatter material. However, MRI is generally only available in a hospital. Also, the flow setup needs to be adjusted so that there are no metallic parts near the MRI-equipment, the help of a laboratory technician is preferred during measurements, and development and adjustment of a protocol or sequence has a large learning curve. The benefit of MRI is the possibility of conversion to a patient study. This translation to a clinical study is not possible in optical PIV, because laser will not pass through the human body and the region of interest inside the patient cannot be captured using digital cameras. Optical PIV is ultimately suitable for accurate and precise quantification of flow patterns *in vitro*. In some situations, another benefit of optical PIV is that a hospital setting is not required to perform the *in vitro* flow studies. Optical PIV models need to be transparent and flat laser-entry surfaces are required for preventing distortion or refraction of the laser beam. Considering the third option, ultrasound, there is a division into studies using clinical systems and studies using research-based systems. To perform detailed flow analysis using image velocimetry techniques, fast imaging is prescribed and therefore the use of a research-based ultrasound system is necessary. However, a research-based ultrasound system has a larger learning curve. Also, research-based systems are not marketed for direct clinical use, so translation to a clinical study is more complicated compared to a clinical system.

**Table 7:** Benefits and limitations of imaging techniques for the design of *in vitro* flow studies.

	MRI	Optical PIV	Ultrasound
Scatter material	**+** Not necessary	**–** Necessary	**–** Necessary
Availability	**–** Only available in hospital	**+** Setup in laboratory-environment (not hospital dependent)	**+** Both in hospital and laboratory-environment
Compatibility setup and models	**–** MR-compatible setup (no metal)	– Limited to transparent and flat-surface models	**–** Need matching acoustic impedance and echogenicity
Easy to use/learning curve	**–** Laboratory technician needed – Learning curve for protocol/sequence development	**+** Relatively small learning curve	**+** Intuitive clinical systems – Learning curve for research systems
Translation to clinical patient study	**+** Possible	**–** No translation possible	**+** Possible – Limited translation possible when using research-based system

In short, MRI and ultrasound are appropriate imaging modalities if translation to a patient study is desired. MRI requires a learning curve or specific knowledge and a metal-free setup. For *in vitro* flow studies, ultrasound requires contrast agents or scatter material in the working fluid. Optical PIV is preferred if there is no aim to translate it to a patient study, if no clinical equipment is available and if restrictions to transparent models are not an issue.

The third step is selection of model materials and model design. This depends on the imaging technique that will be used. For example, poly(vinyl alcohol) gel-based models are ultimately suitable for ultrasound studies. If optical PIV is considered, silicone models are preferred, such as polydimethylsiloxane (PDMS). As stated before, the models for optical PIV need to be transparent and require flat surfaces. Ultrasound models often have a tissue mimicking layer, while MRI-models frequently are thin-walled without TMM. Remarkably, the included studies barely reported on choices made in model selection. For example, only a few studies indicated that the model is elastic or rigid and moreover, elasticity was quantified in only four articles.^9^,^24^,^48^,^49^ These are certainly parameters that need to be considered, since rigidity or elasticity has a large influence on the similarity of the simulation with the real situation.

### Strengths and Limitations of this Review

This review summarizes *in vitro* carotid artery flow studies according to used imaging techniques, model materials and designs. Compared to other review articles which usually focus on one specific imaging technique, this review has a wide scope as it provides an overview of multiple imaging techniques. For the design of an *in vitro* flow study, this wide scope is beneficial, because model materials and model designs depend on the chosen imaging techniques.

In the ultrasound category, multiple articles of the same research group are found. This is a bias to the results which we took into consideration by noting the research groups (Table 3). Furthermore, we marked the flow parameters that we found in multiple articles from the same research group (Table 6). Since our analysis does not strongly depend on the number of parameters and it only happens in the ultrasound category, this bias does not influence our conclusions.

The notations and units of several parameters, such as accuracy, resolution, viscosity, vary widely among the included articles. Therefore, interpretation of these parameters was difficult.

To the authors knowledge, imaging techniques and model parameters might have been missed in our search by restricting it to carotid artery bifurcations. Moreover, newest techniques might be tested first on ‘simple’ straight models, so we might have missed these novel techniques by restricting the search. Techniques and methods might have been missed as well by restricting the search to carotid arteries, since *in vitro* flow studies are also widely performed in intracranial, abdominal or other peripheral artery models. On the other hand, carotid artery specific flow rates, types of diseases, and vessel wall characteristics, lead to specific choices of materials and methods in the design of the flow study.

The imaging techniques reported in this review are not only used to study carotid arteries. Other parts of the cardiovascular system are studied as well. The ascending aorta,^21^,^25^,^62^ aortic arch^3^ and also aortic coarctation^42^ are studied using three- and four-dimensional flow MRI. In addition, this technique is applied intracranially to study aneurysms,^5^ also in combination with optical PIV flow studies.^55^,^61^ Furthermore, ventricular filling is studied using four-dimensional flow MRI.^16^ Ultrasound-based vector flow imaging and echoPIV are used to study flow in the ascending aorta,^22^ the abdominal aorta,^17^,^63^ and to study ventricular blood flow.^27^,^32^ The latter is also reported in combination with optical PIV flow studies.^1^,^64^ Moreover, optical PIV is applied in enlarged coronary artery flow phantoms.^6^,^60^ Thus, despite the restriction of the systematic search to carotid artery bifurcation, the reported imaging techniques and considerations for the design of an *in vitro* flow study can generally be applied.

Fabrication methods of the models for *in vitro* flow studies fall out of the scope of this review article. Only a small amount of the included articles reported about the fabrication method of the models. A specific search to articles concerning the process of constructing models is necessary to write a review article on that topic. Two literature reviews for ultrasound and PIV models specifically have been published already.^13^,^71^

## Conclusion

This systematic review on *in vitro* flow studies aiming at quantifying and visualizing flow parameters in carotid bifurcation models shows important factors to consider when designing a flow study. In contrast to most other review articles on flow studies, this review is not restricted to one imaging modality. Therefore, it gives a complete overview of techniques for *in vitro* flow studies.

Since the design of flow studies strongly depends on the pertinent research question at hand, there is no preferred imaging technique or design that can be selected based on the information in this review. Three important steps need to be considered while designing *in vitro* flow studies: (1) selection of flow visualization parameters, (2) selection of an imaging technique, (3) model materials and design.

The selection of flow visualization parameters is completely dependent on the aim and goal of the study and independent of the selected imaging modality. Flow parameters are classified into velocity, flow, shear-related and turbulent/disordered flow-based parameters. The selection of an imaging technique can roughly be categorized in MRI, optical PIV and ultrasound. Conclusions on accuracy and resolution of the imaging systems cannot be made, since these parameters are not consistently reported throughout the literature. The selection of model materials and design of the model depends on the imaging technique and it strongly depends on the goal of the study.

## References

[1] Asami R, Tanaka T, Kawabata KI, Hashiba K, Okada T, Nishiyama T (2017). Accuracy and limitations of vector flow mapping: left ventricular phantom validation using stereo particle image velocimetory. J. Echocardiogr..

[2] Bale-Glickman J, Selby K, Saloner D, Savas O (2003). Experimental flow studies in exact-replica phantoms of atherosclerotic carotid bifurcations under steady input conditions. J. Biomech. Eng..

[3] Binter C, Gotschy A, Sündermann SH, Frank M, Tanner FC, Lüscher TF (2017). Turbulent kinetic energy assessed by multipoint 4-dimensional flow magnetic resonance imaging provides additional information relative to echocardiography for the determination of aortic stenosis severity. Circ. Cardiovasc. Imaging.

[4] Botnar R, Rappitsch G, Scheidegger MB, Liepsch D, Perktold K, Boesiger P (2000). Hemodynamics in the carotid artery bifurcation: a comparison between numerical simulations and in vitro MRI measurements. J. Biomech..

[5] Boussel L, Rayz V, Martin A, Acevedo-Bolton G, Lawton MT, Higashida R (2009). Phase-contrast magnetic resonance imaging measurements in intracranial aneurysms in vivo of flow patterns, velocity fields, and wall shear stress: comparison with computational fluid dynamics. Magn. Reson. Med..

[6] Brunette J, Mongrain R, Laurier J, Galaz R, Tardif JC (2008). 3D flow study in a mildly stenotic coronary artery phantom using a whole volume PIV method. Med. Eng. Phys..

[7] Buchmann NA, Atkinson C, Jeremy MC, Soria J (2011). Tomographic particle image velocimetry investigation of the flow in a modeled human carotid artery bifurcation. Exp. Fluids..

[8] Carvalho JL, Nielsen JF, Nayak KS (2010). Feasibility of in vivo measurement of carotid wall shear rate using spiral Fourier velocity encoded MRI. Magn. Reson. Med..

[9] Chee AJY, Ho CK, Yiu BYS, Yu ACH (2016). Walled carotid bifurcation phantoms for imaging investigations of vessel wall motion and blood flow dynamics. IEEE Trans Ultrason. Ferroelectr. Freq. Control..

[10] Cheung SC, Wong KK, Yeoh GH, Yang W, Tu J, Beare R (2010). Experimental and numerical study on the hemodynamics of stenosed carotid bifurcation. Australas. Phys. Eng. Sci. Med..

[11] Cibis M, Potters WV, Gijsen FJ, Marquering H, Van Ooij P, Van Bavel E (2016). The effect of spatial and temporal resolution of cine phase contrast MRI on wall shear stress and oscillatory shear index assessment. PLoS ONE..

[12] Couch GG, Johnston KW, Ojha M (1996). Full-field flow visualization and velocity measurement with a photochromic grid method. Measurement Sci. Technol..

[13] Culjat MO, Goldenberg D, Tewari P, Singh RS (2010). A review of tissue substitutes for ultrasound imaging. Ultrasound Med. Biol..

[14] Ding Z, Liu B, Yang S, Xia Y (2008). Hemodynamics for asymmetric inlet axial velocity profile in carotid bifurcation model. J. Hydrodyn..

[15] Ding Z, Wang K, Li J, Cong X (2001). Flow field and oscillatory shear stress in a tuning-fork-shaped model of the average human carotid bifurcation. J. Biomech..

[16] Elbaz MSM, Calkoen EE, Westenberg JJM, Lelieveldt BPF, Roest AAW, Van Der Geest RJ (2014). Vortex flow during early and late left ventricular filling in normal subjects: quantitative characterization using retrospectively-gated 4D flow cardiovascular magnetic resonance and three-dimensional vortex core analysis. J. Cardiovasc. Magn. Reson..

[17] Engelhard S, Voorneveld J, Vos HJ, Westenberg JJM, Gijsen FJH, Taimr P (2018). High-frame-rate contrast-enhanced US particle image velocimetry in the abdominal aorta: first human results. Radiology.

[18] Frayne R, Gowman LM, Rickey DW, Holdsworth DW, Picot PA, Drangova M (1993). A geometrically accurate vascular phantom for comparative studies of x-ray, ultrasound, and magnetic resonance vascular imaging: construction and geometrical verification. Med. Phys..

[19] Gibson CM, Diaz L, Kandarpa K, Sacks FM, Pasternak RC, Sandor T (1993). Relation of vessel wall shear stress to atherosclerosis progression in human coronary arteries. Arterioscler. Thromb..

[20] Gupta A, Baradaran H, Schweitzer AD, Kamel H, Pandya A, Delgado D (2013). Carotid plaque MRI and stroke risk: a systematic review and meta-analysis. Stroke..

[21] Guzzardi DG, Barker AJ, Van Ooij P, Malaisrie SC, Puthumana JJ, Belke DD (2015). Valve-related hemodynamics mediate human bicuspid aortopathy: insights from wall shear stress mapping. J. Am. Coll. Cardiol..

[22] Hansen KL, Møller-Sørensen H, Kjaergaard J, Jensen MB, Lund JT, Pedersen MM (2016). Intra-operative vector flow imaging using ultrasound of the ascending aorta among 40 patients with normal, stenotic and replaced aortic valves. Ultrasound Med. Biol..

[23] He X, Ku DN (1996). Pulsatile flow in the human left coronary artery bifurcation: average conditions. J. Biomech. Eng..

[24] Hewlin RL, Kizito JP (2018). Development of an experimental and digital cardiovascular arterial model for transient hemodynamic and postural change studies: “a preliminary framework analysis”. Cardiovasc. Eng. Technol..

[25] Hope MD, Hope TA, Crook SES, Ordovas KG, Urbania TH, Alley MT (2011). 4D flow CMR in assessment of valve-related ascending aortic disease. JACC Cardiovasc. Imaging.

[26] Howard DPJ, Van Lammeren GW, Rothwell PM, Redgrave JN, Moll FL, De Vries JPPM (2015). Symptomatic carotid atherosclerotic disease: correlations between plaque composition and ipsilateral stroke risk. Stroke..

[27] Itatani K, Okada T, Uejima T, Tanaka T, Ono M, Miyaji K (2013). Intraventricular flow velocity vector visualization based on the continuity equation and measurements of vorticity and wall shear stress. Jpn. J. Appl. Phys..

[28] Jensen J, Hoyos CAV, Traberg MS, Olesen JB, Tomov BG, Moshavegh R (2018). Accuracy and precision of a plane wave vector flow imaging method in the healthy carotid artery. Ultrasound Med. Biol..

[29] Kabinejadian F, Cui F, Zhang Z, Ho P, Leo HL (2013). A novel carotid covered stent design: in vitro evaluation of performance and influence on the blood flow regime at the carotid artery bifurcation. Ann. Biomed. Eng..

[30] Kefayati S, Milner JS, Holdsworth DW, Poepping TL (2014). In vitro shear stress measurements using particle image velocimetry in a family of carotid artery models: effect of stenosis severity, plaque eccentricity, and ulceration. PLoS ONE..

[31] Kefayati S, Poepping TL (2013). Transitional flow analysis in the carotid artery bifurcation by proper orthogonal decomposition and particle image velocimetry. Med. Eng. Phys..

[32] Kheradvar A, Houle H, Pedrizzetti G, Tonti G, Belcik T, Ashraf M (2010). Echocardiographic particle image velocimetry: a novel technique for quantification of left ventricular blood vorticity pattern. J. Am. Soc. Echocardiogr..

[33] Kohler U, Marshall I, Robertson MB, Long Q, Xu XY, Hoskins PR (2001). MRI measurement of wall shear stress vectors in bifurcation models and comparison with CFD predictions. J. Magn. Reson Imaging.

[34] Ku DN, Giddens DP, Phillips DJ, Strandness DE (1985). Hemodynamics of the normal human carotid bifurcation: in vitro and in vivo studies. Ultrasound Med. Biol..

[35] Lai SS, Yiu BY, Poon AK, Yu AC (2013). Design of anthropomorphic flow phantoms based on rapid prototyping of compliant vessel geometries. Ultrasound Med. Biol..

[36] Leow CH, Bazigou E, Eckersley RJ, Yu AC, Weinberg PD, Tang MX (2015). Flow velocity mapping using contrast enhanced high-frame-rate plane wave ultrasound and image tracking: methods and initial in vitro and in vivo evaluation. Ultrasound Med. Biol..

[37] Leow CH, Tang MX (2018). Spatio-temporal flow and wall shear stress mapping based on incoherent ensemble-correlation of ultrafast contrast enhanced ultrasound images. Ultrasound Med. Biol..

[38] Long Q, Xu XY, Kohler U, Robertson MB, Marshall I, Hoskins P (2002). Quantitative comparison of CFD predicted and MRI measured velocity fields in a carotid bifurcation phantom. Biorheology..

[39] Marshall I, Zhao S, Papathanasopoulou P, Hoskins P, Xu Y (2004). MRI and CFD studies of pulsatile flow in healthy and stenosed carotid bifurcation models. J. Biomech..

[40] Mokhtar NH, Abas A, Razak NA, Hamid MNA, Teong SL (2017). Effect of different stent configurations using Lattice Boltzmann method and particles image velocimetry on artery bifurcation aneurysm problem. J Theor. Biol..

[41] Moore JE, Xu C, Glagov S, Zarins CK, Ku DN (1994). Fluid wall shear stress measurements in a model of the human abdominal aorta: oscillatory behavior and relationship to atherosclerosis. Atherosclerosis.

[42] Muzzarelli S, Meadows AK, Ordovas KG, Higgins CB, Meadows JJ (2012). Usefulness of cardiovascular magnetic resonance imaging to predict the need for intervention in patients with coarctation of the aorta. Am. J. Cardiol..

[43] Napel S, Lee DH, Frayne R, Rutt BK (1992). Visualizing three-dimensional flow with simulated streamlines and three-dimensional phase-contrast MR imaging. J. Magn. Reson. Imaging.

[44] Nemati M, Loozen GB, van der Wekken N, van de Belt G, Urbach HP, Bhattacharya N (2015). Application of full field optical studies for pulsatile flow in a carotid artery phantom. Biomed. Opt. Express..

[45] Niu L, Zhu X, Pan M, Derek A, Xu L, Meng L (2018). Influence of vascular geometry on local hemodynamic parameters: phantom and small rodent study. Biomed. Eng. Online..

[46] Papathanasopoulou P, Zhao S, Kohler U, Robertson MB, Long Q, Hoskins P (2003). MRI measurement of time-resolved wall shear stress vectors in a carotid bifurcation model, and comparison with CFD predictions. J. Magn. Reson. Imaging..

[47] Peiffer V, Sherwin SJ, Weinberg PD (2013). Does low and oscillatory wall shear stress correlate spatially with early atherosclerosis? A systematic review. Cardiovasc. Res..

[48] Poepping TL, Nikolov HN, Rankin RN, Lee M, Holdsworth DW (2002). An in vitro system for Doppler ultrasound flow studies in the stenosed carotid artery bifurcation. Ultrasound Med. Biol..

[49] Poepping TL, Nikolov HN, Thorne ML, Holdsworth DW (2004). A thin-walled carotid vessel phantom for Doppler ultrasound flow studies. Ultrasound Med. Biol..

[50] Poepping TL, Rankin RN, Holdsworth DW (2010). Flow patterns in carotid bifurcation models using pulsed Doppler ultrasound: effect of concentric vs. eccentric stenosis on turbulence and recirculation. Ultrasound Med. Biol..

[51] Ramnarine KV, Nassiri DK, Hoskins PR, Lubbers J (1998). Validation of a new blood-mimicking fluid for use in Doppler flow test objects. Ultrasound Med. Biol..

[52] Randomised trial of endarterectomy for recently symptomatic carotid stenosis: final results of the MRC European Carotid Surgery Trial (ECST). *Lancet.* 351(9113):1379–1387, 1998. 10.1016/S0140-6736(97)09292-1. 9593407

[53] Reneman RS, Arts T, Hoeks APG (2006). Wall shear stress—an important determinant of endothelial cell function and structure—in the arterial system in vivo: discrepancies with theory. J. Vasc. Res..

[54] Rispoli VC, Nielsen JF, Nayak KS, Carvalho JLA (2015). Computational fluid dynamics simulations of blood flow regularized by 3D phase contrast MRI. BioMedical Eng. Online.

[55] Roloff C, Stucht D, Beuing O, Berg P (2019). Comparison of intracranial aneurysm flow quantification techniques: standard PIV vs stereoscopic PIV vs tomographic PIV vs phase-contrast MRI vs CFD. J. NeuroInterv. Surg..

[56] Seong J, Jeong W, Smith N, Towner RA (2015). Hemodynamic effects of long-term morphological changes in the human carotid sinus. J. Biomech..

[57] Shaaban AM, Duerinckx AJ (2000). Wall shear stress and early atherosclerosis: a review. Am. J. Roentgenol..

[58] Sheeran PS, Luois S, Dayton PA, Matsunaga TO (2011). Formulation and acoustic studies of a new phase-shift agent for diagnostic and therapeutic ultrasound. Langmuir: ACS J. Surf. Colloids.

[59] Shimizu M, Tanaka T, Okada T, Seki Y, Nishiyama T (2017). Wall shear stress measurement method based on parallel flow model near vascular wall in echography. Jpn. J. Applied . Phys..

[60] Shintani Y, Iino K, Yamamoto Y, Kato H, Takemura H, Kiwata T (2018). Analysis of computational fluid dynamics and particle image velocimetry models of distal-end side-to-side and end-to-side anastomoses for coronary artery bypass grafting in a pulsatile flow. Circ. J..

[61] van Ooij P, Guédon A, Poelma C, Schneiders J, Rutten MCM, Marquering HA (2012). Complex flow patterns in a real-size intracranial aneurysm phantom: phase contrast MRI compared with particle image velocimetry and computational fluid dynamics. NMR Biomed..

[62] Von Knobelsdorff-Brenkenhoff F (2017). Advanced assessment of aortic stenosis reflecting the complex interplay of valve, ventricle, vessel, and flow. Circ. Cardiovasc. Imaging.

[63] Voorneveld J, Engelhard S, Vos HJ, Reijnen MMPJ, Gijsen F, Versluis M (2018). High-frame-rate contrast-enhanced ultrasound for velocimetry in the human abdominal aorta. IEEE Trans. Ultrason. Ferroelectr. Freq. Control..

[64] Voorneveld J, Muralidharan A, Hope T, Vos HJ, Kruizinga P, Van Der Steen AFW (2018). High frame rate ultrasound particle image velocimetry for estimating high velocity flow patterns in the left ventricle. IEEE Trans. Ultrason. Ferroelectr. Freq. Control..

[65] Vu AT, Lee HK, Moran PR, Nalcioglu O (1993). Flow field mapping by multi-zone adiabatic passage excitation. Magn. Reson. Imaging..

[66] Wolf RL, Richardson DB, LaPlante CC, Huston J, Riederer SJ, Ehman RL (1992). Blood flow imaging through detection of temporal variations in magnetization. Radiology..

[67] Wong EY, Nikolov HN, Rankin RN, Holdsworth DW, Poepping TL (2013). Evaluation of distal turbulence intensity for the detection of both plaque ulceration and stenosis grade in the carotid bifurcation using clinical Doppler ultrasound. Eur. Radiol..

[68] Wong EY, Nikolov HN, Thorne ML, Poepping TL, Rankin RN, Holdsworth DW (2009). Clinical Doppler ultrasound for the assessment of plaque ulceration in the stenosed carotid bifurcation by detection of distal turbulence intensity: a matched model study. Eur. Radiol..

[69] Wong EY, Thorne ML, Nikolov HN, Poepping TL, Holdsworth DW (2008). Doppler ultrasound compatible plastic material for use in rigid flow models. Ultrasound Med. Biol..

[70] Xing R, Moerman AM, Ridwan Y, Daemen MJ, van der Steen AFW, Gijsen FJH (2018). Temporal and spatial changes in wall shear stress during atherosclerotic plaque progression in mice. R Soc Open Sci..

[71] Yazdi SG, Geoghegan PH, Docherty PD, Jermy M, Khanafer A (2018). A review of arterial phantom fabrication methods for flow measurement using PIV techniques. Ann. Biomed. Eng..

[72] Yiu BY, Lai SS, Yu AC (2014). Vector projectile imaging: time-resolved dynamic visualization of complex flow patterns. Ultrasound Med. Biol..

[73] Yiu BY, Yu AC (2013). High-frame-rate ultrasound color-encoded speckle imaging of complex flow dynamics. Ultrasound in medicine & biology..

[74] Yoshida K (1986). Blood flow analysis using digital subtraction angiography. Nihon Univ. J. Med..

[75] Yousif MY, Holdsworth DW, Poepping TL (2010). A blood-mimicking fluid for particle image velocimetry with silicone vascular models. Exp. Fluids..

[76] Zarins CK, Giddens DP, Bharadvaj BK, Sottiurai VS, Mabon RF, Glagov S (1983). Carotid bifurcation atherosclerosis. Quantitative correlation of plaque localization with flow velocity profiles and wall shear stress. Circ. Res..

[77] Zhang F, Lanning C, Mazzaro L, Barker AJ, Gates PE, Strain WD (2011). In vitro and preliminary in vivo validation of echo particle image velocimetry in carotid vascular imaging. Ultrasound Med. Biol..

[78] Zhao SZ, Papathanasopoulou P, Long Q, Marshall I, Xu XY (2003). Comparative study of magnetic resonance imaging and image-based computational fluid dynamics for quantification of pulsatile flow in a carotid bifurcation phantom. Ann. Biomed. Eng..

